# Cannabis use in male and female first episode of non-affective psychosis patients: Long-term clinical, neuropsychological and functional differences

**DOI:** 10.1371/journal.pone.0183613

**Published:** 2017-08-23

**Authors:** Esther Setién-Suero, Karl Neergaard, Mariluz Ramírez-Bonilla, Patricia Correa-Ghisays, Lourdes Fañanás, Benedicto Crespo-Facorro, Rosa Ayesa-Arriola

**Affiliations:** 1 University Hospital Marqués de Valdecilla, Department of Psychiatry, School of Medicine, University of Cantabria, Santander, Spain; 2 CIBERSAM, Biomedical Research Network on Mental Health Area, Madrid, Spain; 3 IDIVAL, Valdecilla Biomedical Research Institute, Santander, Spain; 4 Laboratoire Parole et Langage, Aix-Marseille University, Aix-en-Provence, France; 5 Faculty of Psychology, University of Valencia, Valencia, Spain; 6 Departament de Biologia Animal, Facultat de Biologia, Universitat de Barcelona, Institut de Biomedicina de la Universitat de Barcelona (IBUB), Barcelona, Spain; Chiba Daigaku, JAPAN

## Abstract

**Background:**

Numerous studies show the existence of a high prevalence of cannabis use among patients with psychosis. However, the differences between men and women who debut with a first episode of psychosis (FEP) regarding cannabis use have not been largely explored. The aim of this study was to identify the specific sex factors and differences in clinical evolution associated with cannabis use.

**Method:**

Sociodemographic characteristics at baseline were considered in our sample of FEP patients to find differences depending on sex and the use of cannabis. Clinical, functional and neurocognitive variables at baseline, 1-year, and 3-years follow-up were also explored.

**Results:**

A total of 549 patients, of whom 43% (N = 236) were cannabis users, 79% (N = 186) male and 21% (N = 50) female, were included in the study. There was a clear relationship between being male and being a user of cannabis (OR = 5.6). Cannabis users were younger at illness onset. Longitudinal analysis showed that women significantly improved in all three dimensions of psychotic symptoms, both in the subgroup of cannabis users and in the non-users subgroup. Conversely, subgroups of men did not show improvement in the negative dimension. In cognitive function, only men presented a significant time by group interaction in processing speed, showing a greater improvement in the subgroup of cannabis users.

**Conclusion:**

Despite knowing that there is a relationship between cannabis use and psychosis, due to the high prevalence of cannabis use among male FEP patients, the results showed that there were very few differences in clinical and neurocognitive outcomes between men and women who used cannabis at the start of treatment compared to those who did not.

## Introduction

To date, studies of sex differences in the manifestation of psychotic disorders have consistently shown that there are differences between men and women in terms of sociodemographic and clinical characteristics, as well as with both premorbid and cognitive functioning [1–3]. Many studies conclude that women tend to experience a less severe course of illness and better prognosis as they show a later age-at-onset and more affective symptoms than men [1, 2, 4]. Meanwhile, women reached higher functional remission as compared to male participants, with significantly more female participants having achieved recovery at the 1-year follow-up [5].

On the other hand, numerous studies show the existence of a high prevalence of cannabis use among first episode psychosis (FEP) patients and those with schizophrenia [6–10]. This prevalence is well above the norm found in the Spanish general population, according to the National Plan on Drugs [11], and coincides with trends of substance use in the United States [12, 13]. The meta-analysis by Koskinen et al. [7] reports a median lifetime rate of cannabis use disorders at 27.1% in people with a schizophrenia diagnosis compared with only 8% in the general population. However, the impact of cannabis on psychotic symptoms is unclear. Some studies demonstrate significant associations between cannabis use and increased positive symptoms [14, 15] while other studies report no significant associations [16, 17]. In the study of Barrowclough et al. [18] after adjustment for covariates, there were no significant associations between cannabis use and psychotic symptoms (positive or negative) or functioning. In addition, change in cannabis use did not significantly predict change in PANSS symptom measures, nevertheless an increase in cannabis use predicted poorer functioning. Several studies have described the differences that appear between patient cannabis users and patient non-users in terms of socio-demographic, clinical, and neurocognitive profiles. However, only a few studies in patients with FEP and cannabis use have included sex in the study of the hypothesis or considered this a primary study variable [19–22]. For this reason, given the importance of sex differences that have emerged from prior studies of patients with an FEP, the study of sex implications for understanding psychotic disorders that occur with the use of cannabis is warranted.

The main aim of this study was to clarify the prevalence of cannabis use in male and female patients with a first episode non-affective psychosis and to explore the influence of sex in the pattern of substance use. The second purpose of this study was to investigate the long-term (1-year and 3-year) course of the symptoms in these subgroups of patients, as well as the evolution of the cognitive functions (at 3-year). We sought to study the relationship between the course of symptoms and cognition, and cannabis use specific to sex.

We hypothesized that FEP male will consume higher quantities of cannabis than female. Furthermore, cannabis user male and female will present more severe psychotic symptoms and poorer cognitive functioning, and will have worse clinical and functional outcomes than male and female non-users.

## Methods

### Study setting

Data for the present investigation were obtained from a large epidemiological and 3-year longitudinal intervention programme of FEP (Programa Asistencial Fases Iniciales de Psicosis [PAFIP]) conducted at the outpatient clinic and the inpatient unit at the University Hospital Marques de Valdecilla, Santander, Spain. It conformed to international standards for research ethics and was approved by the local institutional review board (NCT02534363). Written informed consent was obtained from all subjects after complete description of the study. When minors were included in PAFIP, parents/legal guardian signed a parental permission consent document. A more detailed descriptions of our programme have previously been reported [23, 24].

### Subjects

All patients included in the current study were assessed over the period between February 2001 and October 2015, and identified as eligible to receive treatment for a first episode of a psychotic disorder under the PAFIP programme.

Patients included in the study were between 15–60 years of age, lived in the catchment area, were experiencing their first episode of psychosis, met the DSM-IV criteria for schizophrenia (50.6%), schizophreniform disorder (28.1%), brief psychotic disorder (11.1%), not otherwise specified (NOS) psychosis (8.4%), schizoaffective disorder (1.5%), or delusional disorder (0.4%) and had no prior treatment with antipsychotic medication or, if previously treated, had a total life time of adequate antipsychotic treatment of less than 6 weeks.

Patients were excluded from the final analysis if they had intellectual disability, brain injury or neurological disease, or had been diagnosed with drug or alcohol dependence according to the DSM-IV criteria.

### Premorbid and sociodemographic variables

Premorbid and sociodemographic information was recorded from patients, relatives and medical records at admission. For the present study we considered: sex, age, years of education, family history of psychosis, hospitalizations, socioeconomic status derived from the parents’ occupations (“low qualification worker” vs. “others”), living area (“urban” vs. “rural”), relationship status (“married/cohabiting” vs. “single/divorced/separate or widowed”), living status (“living with parents” vs. “other”), employment status (“employed” vs. “unemployed”).

For the purposes of this report, patients were grouped according to the presence or absence of cannabis use prior to illness (psychosis) onset, as “users” or “non-users”. Age of cannabis use onset, duration of cannabis use, amount of cannabis used, and classification of subjects as users or non-users, was based on the verbal report by the patients taken during clinical interviews by the clinical team.

### Clinical and neurocognitive variables

The following are the clinical variables considered in the study: duration of untreated illness (DUI), defined as the time from the first unspecific symptoms related to psychosis to initiation of adequate antipsychotic drug treatment (for such a symptom to be considered, there should be no return to previous stable level of functioning); duration of untreated psychosis (DUP), defined as the time from the first continuous psychotic symptoms (present most of the time) to initiation of adequate antipsychotic drug treatment; insight, assessed with the Scale Unawareness of Mental Disorders (SUMD) [25]. The symptoms of psychosis were assessed by mean scores on the Scale for the Assessment of Negative Symptoms (SANS) [26], and the Scales for the Assessment of Positive Symptoms (SAPS) [27]. The SANS and SAPS scores were used in generating dimensions of positive, disorganized and negative symptoms [28]. Depression symptoms were evaluated using the Calgary Depression Scale for Schizophrenia (CDSS) [29]. The schizophrenia diagnoses were confirmed through the use of the Structured Clinical Interview for DSM-IV (SCID-I) [30]. Functional assessment was conducted with The Disability Assessment Scale (DAS) Spanish version [31] and with Global Assessment Functioning (GAF) [32]. The data that comprise the current study were collected at baseline to the program, and at 1 and 3 years post entrance.

A composite metric known as Global Cognitive Functioning (GCF) was also used. Following previously reported transformations [33], it comprises results from tasks representing seven cognitive domains: 1) Verbal Memory, assessed with The Rey Auditory Verbal Learning Test (RAVLT) [34]; 2) Visual Memory, assessed with Rey Complex Figure (RFC) [35]; 3) Working Memory, assessed with WAIS-III digits backward subtest [36]; 4) Executive Function, assessed with Trail Making Test (TMT) [37]; 5) Processing Speed, assessed with WAIS-III digits symbol subtest [36]; 6) Motor Dexterity, assessed with Grooved Pegboard Test [38]; and 7) Attention, assessed with Continuous Performance Test (CPT) [39]. For purposes of the current study baseline and 3-year assessment were considered.

### Statistical analysis

The Statistical Package for Social Science, version 19.0, was used for statistical analyses [40]. The normality of the distribution was assessed using the Kolmogorov-Smirnov test. Parametric (t-test) and nonparametric (Mann-Whitney U) tests were used for comparisons of continuous variables, with Cohen’s d effect size (ES) analysis performed to determine the magnitude of the differences. Categorical variables were compared by using a chi-square test. A repeated measure ANCOVA was performed for clinical, functional, and cognitive variables. Effects of time (longitudinal dimension), group (cross-sectional dimension) and time by group (interaction effect) were examined. All post-hoc comparisons were Bonferroni corrected.

## Results

A total of 549 patients were included in this study: 43% (N = 236) were cannabis users, of which 79% (N = 186) were male and 21% (N = 50) were female. The subgroup of cannabis users was characterized by being significantly younger at the time of entry into the program (u = 17646; p≤0.001). There was a significant relationship between being male and being a cannabis user (OR = 5.6; X^2^ = 82.81; p≤0.001).

### Differences between cannabis users and non-users by sex

#### Differences between male cannabis users and non-users

As observed in **Table 1**, male cannabis users were characterized by having significantly earlier illness onset (t = -6.942; p<0.001), less completed education (u = 9100.5; p = 0.002) and being more often single (X^2^ = 8.375; p = 0.004) than non-users. In clinical and functional variables, cannabis users presented significantly more severe positive [SAPS (u = 9793.5; p = 0.018)] and disorganized [disorganized dimension (u = 9583.5; p = 0.008)] symptoms, but better functioning [DAS (u = 7590; p = 0.001)] than non-users. Concerning cognitive function, cannabis users performed significantly worse in processing speed (t = -2.76; p = 0.006) and better in attention (u = 5001; p = 0.017), showing significant better global cognitive functioning [GCF (t = -2.106; p = 0.037)].

**Table 1:** Differences between cannabis users and non-users by sex.

	Male						Female						Gender comparison	
	Cannabis users		Non-users				Cannabis users		Non-users				Cannabis users	
	N	Mean (SD/%)	N	Mean (SD/%)	p value	Effect size	N	Mean (SD/%)	N	Mean (SD/%)	p value	Effect size	P value	Effect size
SOCIODEMOGRAPHIC VARIABLES														
Age, years	186	24.84 (5.59)	125	31.42 (9.54)	p<0.001**	-0.840	50	27.01 (8.14)	188	35.37 (10.25)	p<0.001**	-0.903	p = 0.206	-0.309
Years of education	184	8.95 (2.68)	123	10.12 (3.29)	p = 0.002**	-0.387	50	10.60 (3.51)	180	11.19 (3.18)	p = 0.243	-0.153	p = 0.002**	-0.550
Familyhistory of psychosis	186	38 (20.43%)	124	30 (24.19%)	p = 0.433		50	15 (30%)	187	44 (23.52%)	p = 0.347		p<0.150	
Hospitalization	186	135 (72.58%)	125	91 (72.8%)	p = 0.966		50	32 (64%)	187	121 (64.70%)	p = 0.926		p<0.236	
Low socio-economic status	179	98 (54.74%)	121	62 (51.23%)	p = 0.550		49	23 (46.93%)	182	99 (54.39%)	p = 0.353		p<0.332	
Urbanarea	183	133 (72.67%)	124	84 (67.74%)	p = 0.351		50	37 (74%)	183	132 (72.13%)	p = 0.793		p<0.852	
Living withparents	184	119 (64.67%)	124	70 (56.45%)	p = 0.146		50	16 (32%)	185	68 (36.75%)	p = 0.533		p<0.001**	
Unmarried	185	169 (91.35%)	125	100 (80%)	p = 0.004**		49	33 (67.34%)	185	95 (51.35%)	p = 0.045*		p<0.001**	
Unemployed	184	93 (50.54%)	124	59 (47.58%)	p = 0.610		50	18 (36%)	185	65 (35.13%)	p = 0.910		p<0.068	
Age of cannabis use onset (years)	164	16.93 (4.10)					46	18.48 (5.42)					p = 0.10	-0.322
Duration of cannabis use	151	8.06 (4.76)					42	8.02 (6.76)					p = 0.962	0.007
Amount (joints/week)	160	27.68 (24.59)					43	18.26 (17.69)					p = 0.004**	0.439
CLINICAL VARIABLES														
DUI (months)	177	18.77 (22.37)	113	27.86 (44.16)	p = 0.206	-0.259	49	14.43 (40.16)	175	23.23 (41.35)	p = 0.242	-0.215	p = 0.025*	0.133
DUP (months)	185	8.61 (12.33)	120	16.08 (32.44)	p = 0.091	-0.304	49	3.73 (4.94)	182	16.48 (37.41)	p = 0.421	-0.477	p = 0.035*	0.522
SAPS	186	14.41 (4.20)	125	13.32 (4.43)	p = 0.018*	0.252	50	13.84 (5.01)	187	13.40 (4.45)	p = 0.542	0.092	p = 0.464	0.123
SANS	184	6.82 (6.34)	125	7.54 (6.69)	p = 0.368	-0.110	50	6.34 (5.51)	185	5.83 (5.74)	p = 0.242	0.090	p = 0.980	0.080
Psychoticdimension	186	7.71 (2.39)	125	7.55 (2.52)	p = 0.483	0.064	50	7.30 (2.38)	187	7.03 (2.51)	p = 0.608	0.110	p = 0.211	0.171
Negativedimension	186	4.92 (5.71)	125	5.95 (5.99)	p = 0.064	0.064	50	4.10 (5.16)	187	4.05 (5.32)	p = 0.596	0.009	p = 0.547	0.150
Disorganizeddimension	186	6.70 (3.40)	125	5.77 (3.39)	p = 0.008**	-0.175	50	6.54 (3.60)	187	6.36 (6.60)	p = 0.425	0.049	p = 0.916	0.045
DAS	168	1.32 (1.52)	115	1.93 (1.59)	p = 0.001**	0.273	45	1.31 (1.32)	167	1.17 (1.42)	p = 0.335	0.101	p = 0.697	0.007
GAF	109	52.11 (32.16)	81	43.79 (28.67)	p = 0.109	-0.390	34	55.35 (29.89)	123	61.36 (31.93)	p = 0.345	-0.194	p = 0.457	-0.104
CDDS	185	2.21 (3.31)	125	2.14 (2.98)	p = 0.984	0.273	50	2.18 (3.01)	187	2.39 (3.29)	p = 0.641	-0.066	p = 0.743	0.009
Schizophrenia diagnosis	186	98 (52.68%)	125	77 (61.6%)	p = 0.120		50	21 (42%)	188	82 (43.61%)	p = 0.837		p<0.180	
SUMD: insight mental illness	167	60 (35.92%)	113	52 (46.01%)	p = 0.091		45	19 (42.22%)	170	83 (48.82%)	p = 0.430		p<0.438	
NEUROCOGNITIVE VARIABLES														
Verbal Memory	137	6.85 (2.89)	101	7.10 (3.20)	p = 0.538	-0.081	39	8.05 (3.51)	149	8.05 (3.33)	p = 0.994	0	p = 0.057	-0.372
Visual Memory	136	19.30 (6.84)	100	17.95 (7.51)	p = 0.153	0.187	39	16.80 (6.47)	147	16.29 (6.56)	p = 0.665	0.078	p = 0.044*	0.374
Working Memory	137	5.50 (1.70)	101	5.47 (1.62)	p = 0.897	0.018	39	5.23 (1.81)	149	5.28 (1.97)	p = 0.925	-0.026	p = 0.285	0.153
Executive Function	133	55.81 (40.10)	99	60.79 (47.24)	p = 0.525	-0.113	36	49.94 (33.21)	142	68 (57.32)	p = 0.158	0.385	p = 0.534	-0.159
Processing speed	137	5.57 (2.62)	100	6.60 (3.10)	p = 0.006**	-0.358	39	7.36 (3)	150	7.93 (2.62)	p = 0.239	-0.202	p<0.001**	-0.635
Motor dexterity	133	71.47 (14.23)	98	79.42 (36.93)	p = 0.131	-0.284	39	66.66 (12.72)	144	72.14 (22.26)	p = 0.120	-0.302	p = 0.060	0.355
Attention	128	72.75 (10.67)	96	66.52 (17.60)	p = 0.017*	0.428	37	68.97 (12.21)	140	67.07 (15.09)	p = 0.619	0.138	p = 0.059	0.329
Premorbid Intelligence (IQ)	132	8.40 (2.74)	99	9.29 (2.99)	p = 0.076	-0.309	39	8.85 (2.46)	146	9.92 (2.38)	p = 0.008**	-0.440	p = 0.365	-0.172
GCF	123	1.38 (0.86)	91	1.67 (1.11)	p = 0.037*	-0.292	34	1.19 (0.84)	129	1.35 (0.94)	p = 0.356	-0.179	p = 0.257	0.223

SAPS: Scale for the Assessment of Positive Symptoms; SANS: Scale for the Assessment of Negative Symptoms; CDSS: Calgary Depression Scale for Schizophrenia; SUMD: Scale Unawareness of Mental Disorders; DUI: Duration of untreated illness; DUP: duration of untreated psychosis; DAS: The Disability Assessment Scale; GAF: Global Assessment Functioning; GCF: Global Cognitive Functioning.

#### Differences between female cannabis users and non-users

The female cannabis users were younger at illness onset (u = 2327.5; p<0.001) and more often single (X^2^ = 4.00; p<0.045) than non-users. Premorbid intelligence resulted significantly different, showing female non-users higher premorbid IQ (u = 2060.5; p = 0.008). Attending clinical and functional variables, no significant differences arose.

#### Differences between male and female cannabis users

We found that males presented significantly longer duration of untreated illness (DUI) (u = 3429; p = 0.025) and duration of untreated psychosis (DUP) (u = 3645; p = 0.035) than females. In the male subgroup there was a higher percentage of patients who lived with their parents than in the female subgroup (X^2^ = 17.196; p<0.001). In addition, the majority of males were single when compared to females (X^2^ = 18.909; p<0.001). Male cannabis users had a lower level of education than female cannabis users (u = 3323; p = 0.002). Regarding neurocognitive variables, men performed significantly better in visual memory (t = 2.03; p = 0.044) and worse in processing speed (t = -3.64; p<0.001) than women. Men used significantly more joints per week than women (u = 2461; p = 0.004).

### Change in symptoms, functioning and cognition over time

#### Cross-sectional between cannabis users and non-users

The results for the clinical variables in patient cannabis users and non-users at baseline, 1-year, and 3-years follow-up assessments are shown in **Table 2**. Significant differences between cannabis users and non-users were found in the male subgroup in symptomatology [negative dimension (F = 4.469; p = 0.036); disorganized dimension (F = 6.389; p = 0.012)] at baseline, showing cannabis users less severe negative symptoms but higher levels of disorganized severity. In function [DAS (F = 7.067; p = 0.008)] male cannabis non-users showed more pronounced functional deficit. In the female subgroup, no significant differences were found between cannabis users and non-users.

**Table 2:** Change in function and symptoms over time.

		Cannabis users			Non-users			F values		
		Baseline	1 year	3 years	Baseline	1 year	3 years	Time	Group	Time x Group
SAPS										
	Male	14.33 (4.29)	2.16 (3.61)	2.82 (4.44)	12.96 (4.41)	1.45 (2.49)	1.58 (2.93)	23.396** a,b	2.135	0.578
	Female	13.76 (4.29)	0.52 (1.61)	1.72 (4.55)	13.44 (4.24)	0.84 (2.03)	0.94 (2.45)	33.101** a,b	0.169	0.359
SANS										
	Male	7.22 (6.48)	5.23 (5.88)	4.83 (5.73)	7.11 (6.45)	5.43 (5.65)	5.48 (6.48)	7.718** b	3.110	0.316
	Female	8 (6.92)	2.92 (4.08)	1.92 (2.43)	6.19 (5.41)	4.49 (5.36)	2.92 (4.39)	5.915** a, c	0.227	1.285
Psychotic dimension										
	Male	7.74 (2.38)	1.44 (2.32)	1.67 (2.66)	7.54 (2.44)	1.08 (1.93)	1.18 (2.30)	19.915** a,b	0.004	0.056
	Female	6.85 (2.92)	0.34 (1.02)	0.73 (1.46)	7.13 (2.39)	0.57 (1.53)	0.51 (1.45)	36.381** a,b	0.426	0.488
Negative dimension										
	Male	5.24 (5.87)	4.75 (5.30)	4.15 (5.05)	5.64 (5.84)	5.17 (5.38)	4.98 (5.99)	3.845*	4.469* f	0.440
	Female	5.68 (6.14)	2.56 (3.57)	1.56 (2.22)	4.02 (4.90)	3.98 (4.90)	2.52 (3.92)	3.312* d,e	0.164	1.866
Disorganized dimension										
	Male	6.58 (3.36)	0.72 (1.68)	1.15 (2.43)	5.38 (3.42)	0.36 (0.96)	0.38 (1.04)	12.081** a,b	6.389* g	1.916
	Female	6.80 (2.94)	0.16 (0.62)	0.96 (3.33)	6.33 (3.51)	0.28 (0.83)	0.43 (1.31)	12.621** a,b	0.834	0.339
DAS										
	Male	1.38 (1.55)	1.58 (1.49)	1.46 (1.42)	1.72 (1.55)	1.88 (1.46)	1.68 (1.56)	2.015	7.067** h	0.242
	Female	1.13 (1.12)	1.17 (1.09)	0.88 (1.91)	1.08 (1.36)	1.35 (1.42)	0.84 (1.22)	1.640	0.077	0.471
GAF										
	Male	51.48 (31.52)	74.71 (23.93)	73.53 (25.44)	47.44 (28.09)	71.87 (23.99)	72.35 (25.78)	0.712	0.525	0.148
	Female	57.12 (28.36)	80.06 (13.30)	87.12 (12.77)	63.99 (30.81)	79.84 (19.05)	83.27 (17.68)	1.593	0.566	1.541

SAPS: Scale for the Assessment of Positive Symptoms; SANS: Scale for the Assessment of Negative Symptoms; DAS: The Disability Assessment Scale; GAF: Global Assessment Functioning

*: p<0.05

**: p<0.01.

Covariates were age and years of education.

a. Users have more symptoms at baseline than at 1-year and at 3-years

b. Non-users have more symptoms at baseline than at 1-year and at 3-years

c. Non-users show differences at all times

d. Users have more symptoms at baseline than at 3-years

e. Non-users have more symptoms at baseline than at 3-years, and at 1-year than 3-year

f. At baseline non-users have more symptoms than users

g. At baseline users have more symptoms than non-users

h. At 1-year users show better function than non-users.

Regarding cognitive function, the male subgroup significantly differed in motor dexterity (F = 5.212; p = 0.025), attention (F = 7.168; p = 0.009) and global cognitive function (F = 9.948; p = 0.002). After Bonferroni correction, male cannabis users significantly outperformed non-users in attention at baseline (F = 4.524; p = 0.036) and at 3-year follow-up (F = 6.783; p = 0.011), showing also better GCF at baseline (F = 4.987; p = 0.028) and at 3-year follow-up (F = 10.108; p = 0.002) than non-users. In motor dexterity male cannabis users significantly outperformed non-users only at 3-year follow-up (F = 4.606; p = 0.034). No significant differences were observed in the female subgroup (See **Table 3**).

**Table 3:** Change in cognition over time.

		Cannabis users		Non-users		F values		
		Baseline	3 years	Baseline	3 years	Time	Group	Time x Group
Verbal Memory								
	Male	6.82 (2.94)	7.97 (3.26)	6.94 (3.08)	7.70 (3.41)	0.379	0.323	0.387
	Female	8.87 (3.64)	9.73 (3.03)	8.25 (3.17)	9.62 (3.03)	3.725	0.421	1.110
Visual Memory								
	Male	19.54 (7.01)	22.08 (7.10)	18.38 (7.78)	21.26 (6.66)	0.343	2.083	0.100
	Female	16.33 (7.90)	17.47 (6.43)	16.37 (6.65)	19.17 (5.84)	0.137	0.372	0.653
Working Memory								
	Male	5.45 (1.90)	5.66 (1.67)	5.55 (1.73)	5.85 (1.78)	0.185	0.712	0.008
	Female	5.00 (1.46)	5.00 (1.46)	5.37(1.96)	5.53 (1.73)	0.519	0.749	0.286
Executive Function								
	Male	-56.77 (43.91)	-49.13 (40.42)	-59.72 (45.92)	-52.68 (45.24)	1.479	1.107	0.015
	Female	-58.07 (41.54)	-43.43 (29.59)	-69.75 (63.58)	-47.14 (47.32)	4.343*^a^	0.068	0.524
Processing speed								
	Male	5.31 (2.60)	6.95 (3.04)	6.67 (3.31)	7.10 (2.64)	0.366	0.190	8.814*
	Female	6.67 (3.56)	9.00 (3.46)	7.70 (2.56)	9.18 (2.94)	2.297	0.567	0.565
Motor dexterity								
	Male	-72.29 (16.20)	-66.48 (14.36)	-78.65 (41.83)	-70.30 (19.67)	3.967*^a^	5.212*^b^	0.941
	Female	-74.00 (16.91)	-62.43 (11.13)	-72.85 (23.94)	-67.12 (17.04)	3.677	0.035	0.444
Attention								
	Male	72.17 (11.90)	74.29 (10.15)	67.67 (16.02)	69.59 (14.16)	0.172	7.168*^c^	0.031
	Female	72.33 (8.91)	76.00 (6.73)	65.73 (16.35)	71.24 (11.16)	0.075	1.441	0.053
Global Cognitve Functioning								
	Male	1.40 (0.83)	1.22 (0.89)	1.65 (1.15)	1.69 (1.11)	0.318	9.948*^c^	1.151
	Female	1.27 (1.15)	1.08 (1.34)	1.34 (0.89)	1.20 (0.99)	0.993	0.034	0.097

*: p<0.05.

**: p<0.01.

Covariates were age, years of education and premorbid IQ.

a. Non-users show better performance at 3-year than at baseline

b. Users outperform non-users at 3-year

c. Users outperform non-users at baseline and 3-year.

### Longitudinal course

As observed in **Table 2**, the male subgroup showed significant improvement in clinical variables [SAPS (F = 23.396; p≤0.001), SANS (F = 7.718; p = 0.001), psychotic dimension (F = 19.915; p≤0.001), negative dimension (F = 3.845; p = 0.022), disorganized dimension (F = 12.081; p≤0.001)] but not in function (DAS and GAF). Post-hoc analyses revealed changes in SAPS, SANS, psychotic dimension and disorganized dimensions, between baseline and 1-year, and between baseline and 3-year follow-up assessments, for both users and non-users (all p<0.05). No significant differences were found between 1-year and 3-year follow-up.

In the female subgroup, the main effects of time were significant across all clinical variables [SAPS (F = 33.101; p≤0.001), SANS (F = 5.915; p = 0.003), psychotic dimension (F = 36.381; p≤0.001), negative dimension (F = 3.312; p = 0.038), disorganized dimension (F = 12.621; p≤0.001)], but not in function. Post-hoc analyses revealed an improvement in SAPS, psychotic and disorganized dimensions between baseline and 1-year, and between baseline and 3-year follow-up assessments, in users and non-users (all p<0.05). Concerning negative symptoms, female cannabis non-users showed significant improvement in SANS at all time points, and in negative dimension between baseline and 3-years, and 1 to 3-year follow-up assessments. Cannabis users showed significant differences in improvement in SANS between baseline and 1-year, and between baseline and 3-year in SANS and in negative dimension. Finally, despite the lack of significant differences in repeated measures analysis, Bonferroni correction revealed significant improvement in DAS between 1-year and 3-year in non-users, and in GAF between baseline and 1-year, and baseline and 3-year follow-up assessments, in users and non-users (See **Table 2**).

With regard to the cognitive variables, the male subgroup showed significant improvement in motor dexterity between baseline and 3-year follow-up assessment (F = 3.967; p = 0.049). Post-hoc analyses revealed an improvement in non-users (F = 6.964; p = 0.010). The female subgroup showed as well significant improvement in executive function (F = 4.343; p = 0.040). Similar to males, after Bonferroni correction, the improvement was confirmed just in the non-users subgroup (F = 13.800; p≤0.001).

The repeated measures analyses did not reveal any significant time by group interaction in clinical and functional variables, between cannabis users and non-users, for either men or women (See **Table 2**). In terms of cognitive function, one significant time by group interaction was found for processing speed in the male subgroup. The magnitude of the improvement was greater in the subgroup of cannabis users than in non-users. (F = 8.814; p = 0.004) (See **Table 3**).

## Discussion

Our results show that sex and age are related to the use of cannabis in first psychotic episode patients. We found differences between male and female cannabis users in cross-sectional analyzes, as well as some differences in the clinical outcome, which are discussed below.

### Cross-sectional differences between subgroups

Young men were more often users of cannabis. At the same time, cannabis users, both male and female, debuted with symptoms of the disease earlier than non-users. These characteristics of cannabis users are consistent with findings from previous studies [15, 41–44], in which being a young male and an early onset of psychosis were significantly associated with cannabis use, [45, 46]. This could be interpreted in two ways: on the one hand, cannabis use somehow precipitates the disease for those individuals genetically vulnerable to the disease, while on the other hand, an early onset of symptoms connotes a search for self-medication as a method to cope with the disease [47–49]. The self-medication hypothesis has not been supported in previous studies [8, 50].

Whereas some studies did not show significant differences between FEP cannabis users and non-users in the number of years of formal education [14, 51], our sample showed that non-users were significantly more likely to achieve a higher education in the male subgroup, but not so in the female subgroup. In a study carried out in Australia with a sample of 6000 subjects from the general population [52], they examined the relationship between the age of onset of cannabis use and educational performance. They found a significant association between the age of onset and the years of education, so that achievements were higher for subjects who had not used cannabis before 18 and lower for those who started consuming before the age of 15. This same article showed a trend which suggests that cannabis use has more detrimental effect on men than women on education, supporting an earlier study [53] which said that the effects of cannabis use on education level may be specific of sex, showing more effects on male academic performance than female. Future studies taking into account the age of onset of cannabis use are warranted to shed light on the possible relationship between age at first exposure to cannabis and the level of education reached, and also to check differences between male and female.

Regarding clinical variables, our sample showed significant differences between users and non-users of cannabis on scores of SAPS and the disorganized dimension, but these differences only appear in the male subgroup. In both cases, cannabis users presented higher scores, which is equivalent to the presence of more severe symptoms. These data are contrary to previous studies that did not show differences in symptoms between cannabis users and non-users [54–57]. However, except in the study of Rabin et al. [57], the samples were not divided by sex, so that the difference in results may be due to the influence exerted in these studies by the female group.

Interestingly and contrary to what we would have expected, the Global Cognitive Functioning of cannabis users showed better overall performance in the male subgroup. However, by examining the cognitive domains separately, we see differences only in attention and processing speed. Patient users had better performance in attention and worse in processing speed than non-users. Several studies that compared global cognition between cannabis users and non-users have shown no significant difference between the two subgroups according to global cognition [58]. Moreover, in a meta-analysis of Yücel et al. [59], the results differed depending on whether comparisons were made between the non-user group and with patients with a history of cannabis use or with current/recent users. The results showed that better cognitive performance is seen only in lifetime users but not in recent users.

### Longitudinal changes in symptomatology and functionality

As observed in other studies, all the subgroups analyzed in this study showed improvement of both positive and negative symptoms at follow-up assessments [19]. After Bonferroni correction, in the female subgroup, this improvement was significant in all three dimensions of psychotic symptoms, in the subgroup of cannabis users and in the non-users subgroup. However, the male subgroup did not show improvement in the negative dimension. These data coincide with results obtained in another study, showing men after two years had a less pronounced improvement than women in negative symptoms [60].

In several studies it has been observed that men present more severe symptoms than women. However, previous studies did not investigate the effect of cannabis use on symptoms [61, 62]. The only study found to refer to the effect of drug use on long-term symptoms is that of Thorup et al. [63] who states that men are more susceptible to negative symptoms than women, regardless of substance use or non-use. The FEP male patients present a defined pattern, with more negative symptoms at all assessments than females and with an evolution less favorable than that found with females. This is a fact of great importance since, as Malla et al. [64] showed in his systematic review, negative symptoms are predictors of poor disease progression.

We found that functioning, measured with scores of DAS and GAF, despite not showing significant differences in repeated measures analysis, Bonferroni correction revealed improvement over time in all subgroups, with the exception of scores on the DAS scale in the male subgroup, in which we found that male cannabis users showed a slight worsening of social functioning after 3 years, while in the non-users subgroup a small improvement appeared. Conversely, female substance users and non-users had better social functioning than males at all time points, a fact that coincides with previous studies [65].

**Fig 1:**
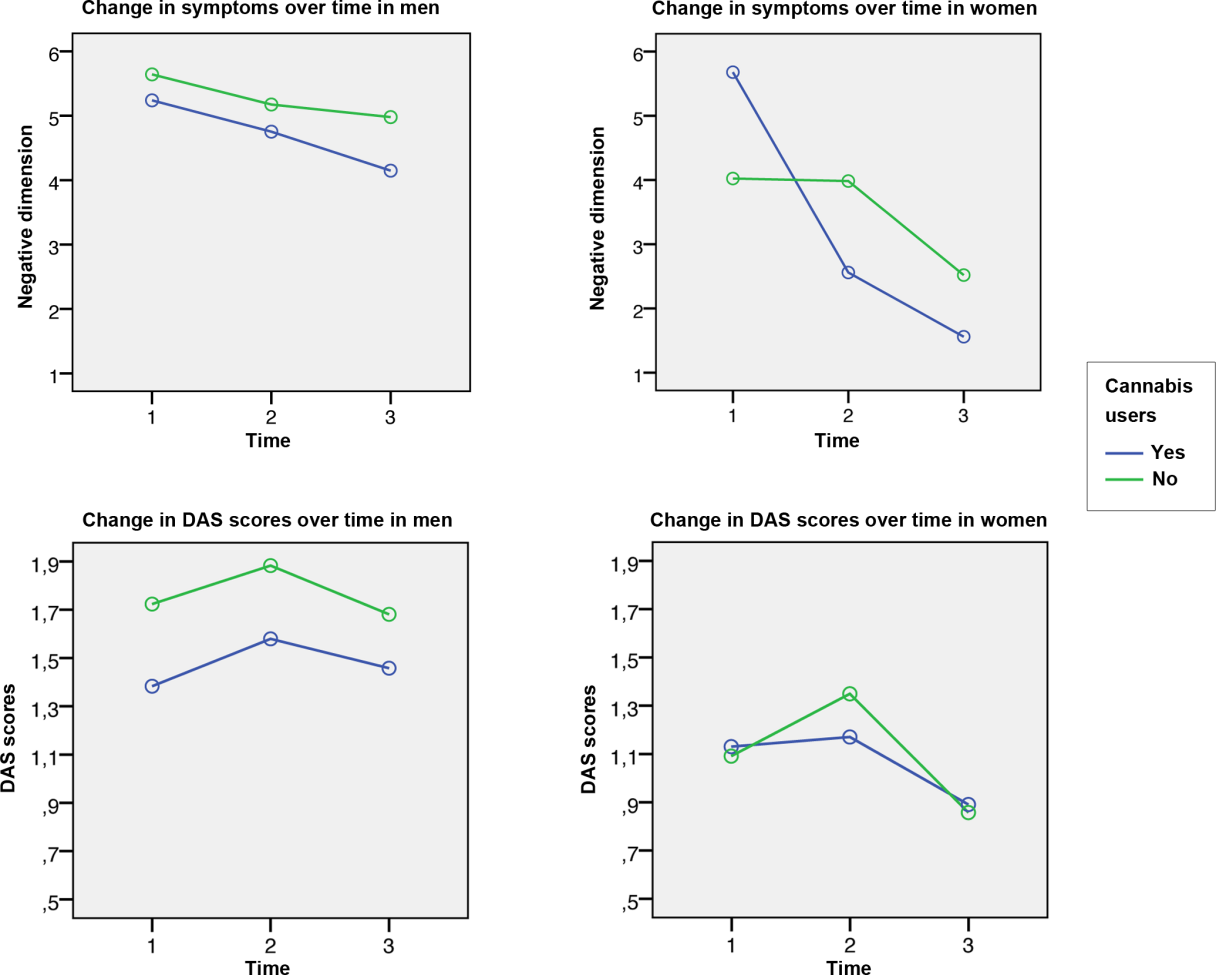
Change in symptoms and functionality over time. DAS: The Disability Assessment Scale.

These results lead us to believe that the poor evolution of the male subgroup according to the negative dimension of the symptoms could be related to the poor evolution of functionality measured with the DAS scale (See **Fig 1**). This would support the idea that more severe symptoms predict lower functioning and higher disability, as shown in the Usall et al. study [65] in which there was a significant association between functionality and negative symptoms in their male group.

As for the evolution of cognitive functions, we found few differences between the subgroups. Specifically, we found a significant interaction of time by group in the processing speed domain, only in the male subgroup. Processing speed is one of the domains most frequently reported as significantly impaired in a large body of schizophrenia studies [66]. However, among patients with schizophrenia, those who are cannabis users show better performance than non-users. In the Yücle et al. meta-analysis [59], eight studies examined the speed of processing, in which it was observed that schizophrenia patient cannabis users had significantly faster processing speeds than patient non-users. In the same study, when recent use and lifetime defining studies was examined separately, patient cannabis users performed significantly better than non-users only within the lifetime defining studies, but not in those studies with recent use criteria. These data are contrary to the ones found in our sample, in which, despite the fact that male cannabis users showed a greater magnitude of improvement than non-users, the cross-sectional analysis showed that there were no significant differences between the users and the non-users, neither at baseline nor at 3-year follow-up assessment.

To summarize, longitudinally there were changes in almost all clinical variables in the subgroups of patients over time among both male and female (except for male negative symptoms). However, there were no differences in the evolution between patients who were cannabis users at the onset of the disease and those who were not. On the other hand, regarding cognitive functions and functionality, patients, both male and female, users and non-users, have negligible changes over time.

### Strengths and limitations

The main strengths of this study lie in its sampling (N = 549) and long-term design, taking into account sex and cannabis use in FEP patients. However the study has several limitations. Firstly, the classification of patients into users or non-users was based on interviews. No confirmation by toxicological urinalysis was performed. However, information and self-reports given by subjects tend to be relatively accurate [67–69]. It should be noted that patients enrolled in our program go through a process in which both clinical and behavioral data are collected from both themselves and their relatives. We thus feel confident as to the utility of self-report measurement of substance use in our sample. Secondly, the number of women who use cannabis is well below the number of male cannabis users. This is consistent with most studies conducted thus far. A third limitation is that the quality and quantity of cannabis used has not been taken into account. The type of cannabis used by patients is unknown, and the classification was done according to being present or absent regardless of the intensity of use, nor the strength of the drug used. Finally, the fourth limitation was the lack of knowledge about the time elapsed since the last use of cannabis until the time of the interview, which could directly affect the psychotic symptomatology and functionality.

To conclude, there is evidence that cannabis use is associated with an earlier onset of psychotic illness. This strongly suggests that the reduction of substance use among high-risk groups could have a significant effect on the incidence of psychosis. However, cannabis use appears to have no effect on psychotic symptoms over time, and to have very little effect on cognitive functions, indicating that it could not influence the course of the disease. There are few studies that discuss the connection that cannabis use may have with psychotic disorders depending on sex. Further studies are needed to elucidate the true relationship between psychosis and cannabis use according to sex.

## Supporting information

S1 File Database 1. (SAV)

S2 File Database 2. (SAV)
